# Impacts of algae supplements (*Arthrospira* & *Chlorella*) on growth, nutrient variables, intestinal efficacy, and antioxidants in New Zealand white rabbits

**DOI:** 10.1038/s41598-023-34914-1

**Published:** 2023-05-16

**Authors:** Mohammed F. El Basuini, Ahmed A. A. Khattab, Salma H. Abu Hafsa, Islam I. Teiba, Nabila E. M. Elkassas, Emad H. El-Bilawy, Mahmoud A. O. Dawood, Safaa Elsayed Salah Atia

**Affiliations:** 1 Faculty of Agriculture, Tanta University, Tanta, 31527 Egypt; 2 King Salman International University, South Sinai, 46618 Egypt; 3 Livestock Research Department, Arid Lands Cultivation Research Institute, City of Scientific Research and Technological Applications, New Borg El-Arab, 21934 Alexandria Egypt; 4 Animal Production Research Institute, Agricultural Research Center, Dokki, Giza, Egypt; 5 Department of Animal Production, Faculty of Agriculture, Kafrelsheikh University, Kafr El-Sheikh, 33516 Egypt; 6 The Center for Applied Research On the Environment and Sustainability, The American University in Cairo, Cairo, 11835 Egypt

**Keywords:** Applied microbiology, Animal physiology

## Abstract

An 8-week trial to examine the impacts of *Arthrospira platensis* and *Chlorella vulgaris* on the growth, nutrient aspects, intestinal efficacy, and antioxidants of 75 New Zealand white male rabbits (initial body weight = 665.93 ± 15.18 g). Herein the study was designed in one-way ANOVA to compare the effects of the two algae species with two levels of supplementations in the feeds of New Zealand white rabbits. The rabbits were divided into five groups (n = 15/group), where the first group was allocated as the control group (Ctrl) while the second and third groups received *A. platensis* at 300 or 500 mg/kg diet (Ap300 or Ap500). The fourth and fifth groups fed *C. vulgaris* at 300 or 500 mg/kg diet (Ch300 or Ch500). The basal diet rabbits exhibited the lowest values of weight, lipase, protease, and the highest feed conversion ratio, which improved noticeably with algae addition, particularly with Ap500, Ch300, and Ch500. All tested groups showed normal intestinal structure. Amylase potency, hematological indicators, and serum biochemistry revealed non-significant variation except for a higher serum total protein and lower total cholesterol in algal groups. The best GPx existed in groups fed algal diets, while favorable SOD and CAT efficiency occurred at the higher level of *Arthrospira* and both levels of *Chlorella*. In conclusion, incorporating *Arthrospira* or *Chlorella* in the diet of New Zealand white rabbits improved performance, nutrient utilization, intestinal efficacy, and antioxidants. *Arthrospira* (Ap500) and *Chlorella* (Ch300 or Ch500) have almost the same beneficial effect on rabbit performance.

## Introduction

Rabbits are one of the most profitable agricultural sectors involved in offering high-quality animal products with distinct merits, remarkably rapid growth, and sexual maturity with high fertility as well as a high gain of meat in carcass^1^. Finding promising tactics to ameliorate organisms' wellbeing and performance is vital for animal production sectors, specifically under stressful circumstances, and the foundation of success is efficient management^2,3^. Antibiotics were broadly employed as growth promoters, stress relievers, and remedies^4^. The usage of antibiotics as growth stimulants in animal production has been banned since 2006 within the European Union^5^. Antibiotics and other synthetic substances have been phased out in favor of more ecologically friendly methods for enhancing animal health, performance, and, eventually, ensuring the safety and superior quality of animal products^6^.

The foundation for good growth is a well-balanced diet, and one of the most effective approaches to altering an animal's growth is to modify the diet^7^. Utilizing functional feed supplements has become a commonly recognized approach for boosting the performance of animals^8^. Algae has the leverage to be a sustainable fountain of food and energy in the future. The majority of microalgal constituents are carbohydrates, lipids, proteins, minerals, vitamins, and bioactive substances^9^. Algal products in the diet of animals have been demonstrated to boost performance and meat goodness in ruminants and nonruminants. These results are greatly reliant on the form of microalgae and their level in the diet^9^.

*Arthrospira* (formerly *Spirulina*) and *Chlorella* are the two genera of algae that warrant a more thorough examination for nutritional purposes. Most microalgae protein fractions are stated to have the same or even better quality than typical plant protein fractions^10^. *Arthrospira* is well-known as a high-protein basis (60–70% of dry weight) with a high digestibility coefficient, with all essential amino acids accounting for about half of total protein^11^, essential fatty acids^12^, phytopigments (carotene—phycocyanin—phycocyanobilin chlorophyll and xanthophyll)^13,14^, water and lipid-soluble vitamins (B group, ascorbic acid, A, D, E, K) as well as minerals (Ca, Cr, Cu, Fe, K, Na, P, Se, Zn)^15^. Dry *Chlorella* has a 50–60% protein content, making it comparable to other sources, e.g., yeast, soy flour, and milk^16^. Also, *Chlorella* biomass provides basic nutrient, pigments, minerals, vitamins, and provitamins^17^. Moreover, dry *Arthrospira* and *Chlorella* microalgae contain a significant portion of lipids (up to 80%) and carbohydrates (12–57%)^16^. *Arthrospira* and *Chlorella* have been proposed as primary ingredients or dietary supplements to enhance the performance and health of animals. In this sense, rabbits treated with *Arthrospira* exhibited higher growth^18–23^, meat quality^18,24^, reproductive performance^25^, immunity^18,19,26,27^, and antioxidants^18,19,21,28,29^. Likewise, rabbits treated with *Chlorella* showed better growth^30–32^, immunity^30,33^, and antioxidants^30,32^.

Considering the high nutritional value of algae, the purpose of the current trial was to contrast the impacts of dry *Chlorella vulgaris* and *Arthrospira platensis* as dietary supplements on the growth, nutrient efficiency, intestinal health, blood indices, and antioxidant capacity in New Zealand white rabbits.

## Materials and methods

### Isolation of algal species

Two algal species were used in this study, namely*, Chlorella vulgaris* and *Arthrospira platensis.* The green alga* Chlorella vulgaris* was isolated from a site in the Damietta branch (Drainage of sewage Omar Buck for 10 km in the city of Mansoura), while the cyanobacterial *Arthrospira platensis* species was isolated from wadi-elnatrun brackish ponds. The isolated algae were developed primarily in a 250-ml Erlenmeyer conical container comprising 100 ml of growth media. For the growth of *Chlorella vulgaris*, Bold`s Basal Medium (BBM) with a final pH of 6.3 was used, while *A. platensis* was enriched in spirulina medium. Unialgal strains were acquired by picking up the clonal population from an algal medium agar plate which was obtained by serial dilution of the primary inoculum.

### Morphological Identification of algal species

**Figure 1:**
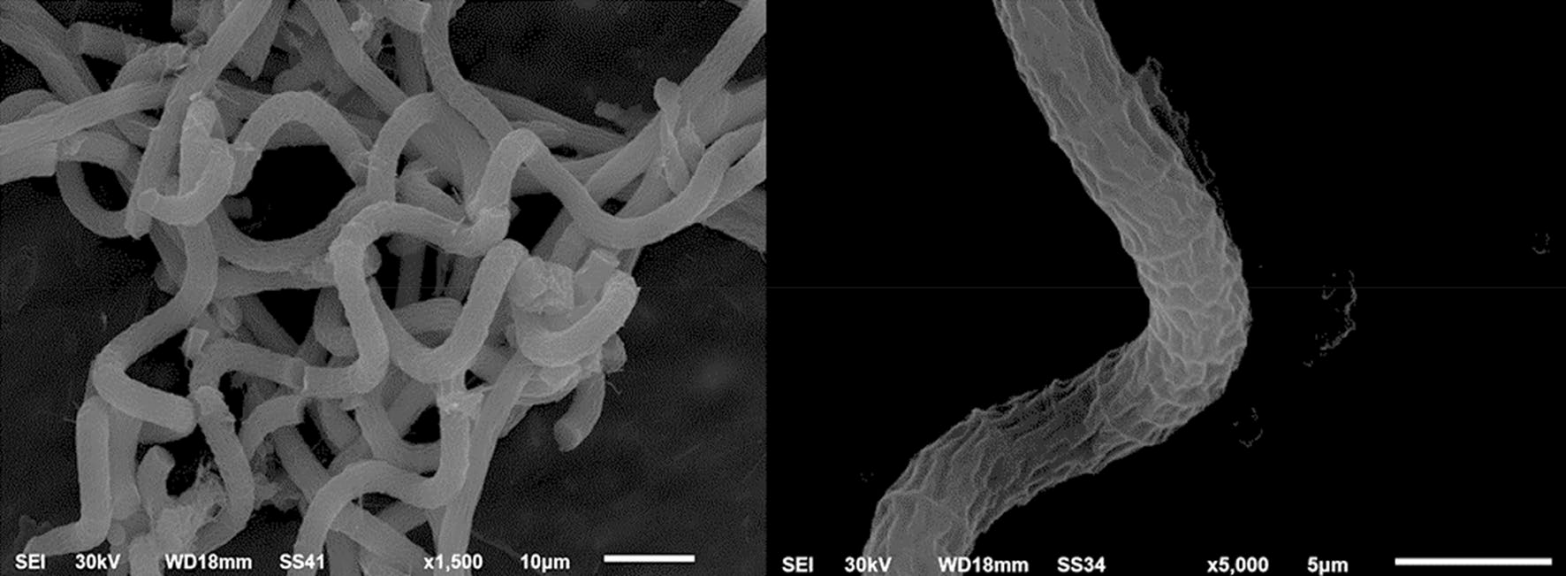
Electron micrograph of *Arthrospira platensis.*

**Figure 2:**
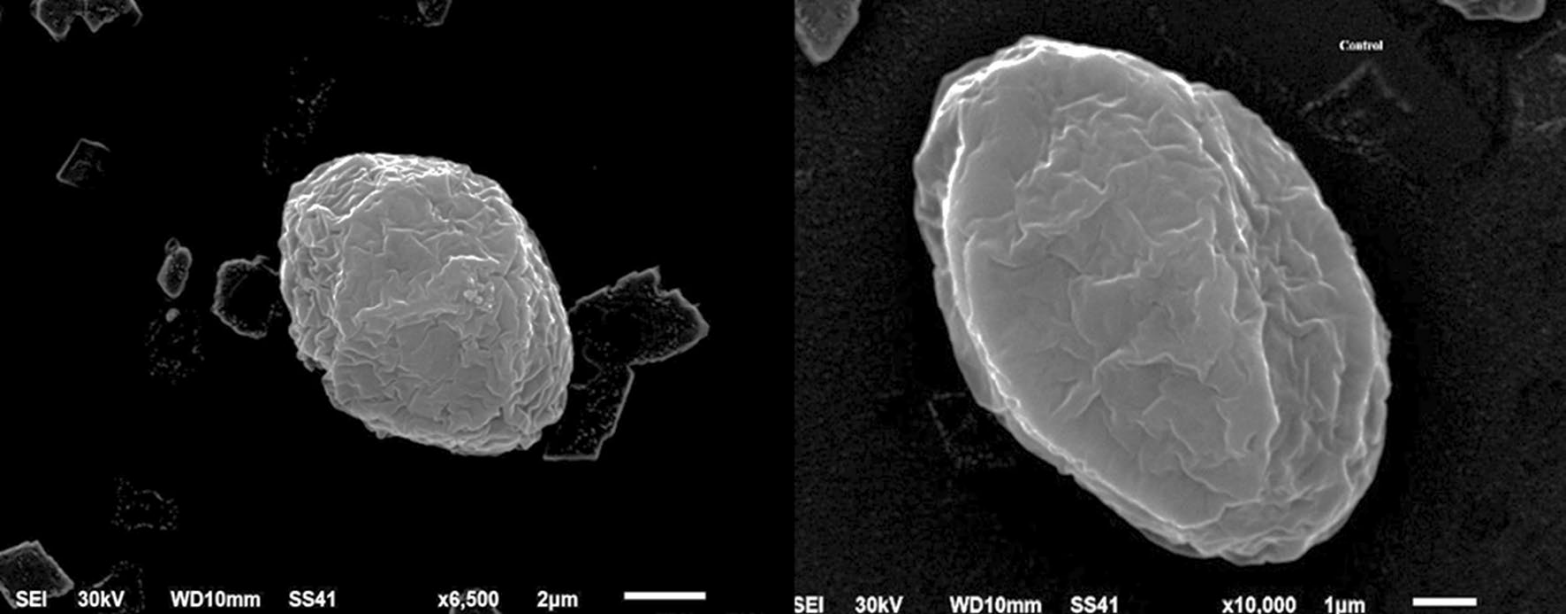
Electron micrograph of *Chlorella vulgaris*.

The isolated algal species were identified morphologically according to features described by Deyab et al.^34^ using a Zeiss (Axiolab 5) light microscope. For more accurate morphological characterization, the isolated species were examined using a JEOL JSM 6510 scanning electron microscope (Figs. 1 and 2).

### Cultivation of algal species

All culture media were incubated on an orbital shaker (130 rpm) at 25 ± 2 °C, with a light intensity of 1.2 Klux, and an illumination of 16:8 h for a week with continuous aeration. The separated algal species were grown in a 2L flask, each comprising 1000 ml of medium, and developed under similar circumstances for 21 days to achieve biomass. To obtain dried biomass, thin layers of wet biomass of both algal species were dehydrated using a Binder Hot oven at 60 °C for 12 h. The chemical content of the tested algae supplements was evaluated following standard analysis techniques^35^.

### Chemical profiling of the algal extract using GC–MS

For the extraction of algae, 1 g of freeze-dried biomass for each alga was extracted twice using 10 ml methanol according to Deyab et al.^34^. To obtain cell-free supernatant, the extracts were centrifuged at 6000 rpm for 20 min, then concentrated using a rotary evaporator at 40 °C. The dried residues were redissolved using 3 ml of methylene chloride and kept at 4 °C until GC–MS analysis. The crude extracts were analyzed using Varian GC–MS (Varian Chrompack CP-3800 GC/MS/MS-2000, Germany). The GC–MS was equipped with a split-splitless injector in addition to a DB-5.625 GC column (30 m × 0.25 mm i.d., 0.25 µm film thickness). The active chemical compounds were identified by matching their recorded spectra with the data bank mass spectra (Saturn and NIST library databases) provided by the instrument software. The concentration (% content) of the components of the extract was computed by integrating their peak areas in the total ion current (TIC) chromatograms, assuming a unity response by all components.

### Animals and management

The experiment was conducted for 8 weeks from December 2020 to January 2021 on a private farm under the supervision of the Animal Production Department, Faculty of Agriculture at Tanta University in Egypt. The ethical committee of the Faculty of Agriculture at Tanta University approved the experimental protocol and all methods in the present study for treating animals for scientific purposes (Approval No. AY_2019-2020_/Session 6/2020.01.13). All experiments were performed in accordance with relevant guidelines and regulations. Our reporting of research involving animals follows the recommendations of the ARRIVE guidelines. Seventy-five five weeks old New Zealand white male rabbits were chosen for litter weight at weaning (665.93 ± 15.18 g) and placed into five experimental groups (n = 15/group). All rabbits were maintained separately in galvanized wire pens (35 × 35 × 60 cm) with freely accessible feeders (ad libitum) and a freshwater outlet under the same management and hygienic conditions, namely a regimen of 12 h light and 12 h dark, natural ventilation, an average temperature of 17.29 ± 0.27 °C and a relative humidity of 59.96 ± 0.42 (Table 1).

**Table 1:** Maximum, minimum, average temperatures, and relative humidity during the experiment (December 2020–January 2021).

Period (December–January)	Temperature °C			Humidity%
	Maximum	Minimum	Average	
1st week	23.29 ± 0.71	14.57 ± 0.20	18.93 ± 0.43	61.71 ± 0.87
2nd week	23.14 ± 0.86	14.57 ± 0.87	18.86 ± 0.68	62.29 ± 0.75
3rd week	21.29 ± 0.29	12.86 ± 0.40	17.07 ± 0.28	60.57 ± 0.65
4th week	20.43 ± 0.43	11.14 ± 0.70	15.79 ± 0.39	61.29 ± 0.71
5th week	21.57 ± 0.48	12.43 ± 0.87	17.00 ± 0.64	59.86 ± 1.06
6th week	24.29 ± 0.87	14.29 ± 0.57	19.29 ± 0.63	61.43 ± 0.78
7th week	20.29 ± 0.64	13.43 ± 0.65	16.86 ± 0.64	58.43 ± 0.84
8th week	19.43 ± 0.61	9.71 ± 0.42	14.57 ± 0.35	54.14 ± 0.59

The rabbits in the reference group (Ctrl) were fed a basal diet with no additions (Table 2), whereas the remainder of the groups were provided a basal diet with 300 or 500 mg of *Arthrospira platensis* (Ap300 or Ap500) or *Chlorella vulgaris* (Ch300 or Ch500).

**Table 2:** The basal diet ingredients and chemical analysis.

Ingredients	%	Chemical analysis	Dry Matter Basis
Yellow corn	18	Crude protein (CP)	17.3%
Soybean meal (44%)	15.1	Ether extract (EE)	2.31%
Barley grain	13	Crude fiber (CF)	13.52%
Berseem hay	33	Ash	3.71%
Wheat bran	16.5	DL-Methionine	0.4%
Molasses	2	Lysine	1.03%
Limestone	1.1	Calcium	0.94%
Dicalcium Phosphate	0.5	Total phosphorus	0.45%
Premix*	0.3	Digestible energy	2530 kcal/kg
DL-Methionine	0.1		
Common salt	0.4		
Total	100		

* Premix provided each kg of feed with Biotin = 0.05 mg; Choline = 250mh; Co = 0.1 mg; Cu = 5 mg; Fe = 50 mg; Folic acid = 3 mg; I = 0.2 mg; Mn = 85 mg; Niacin = 50 mg; Pantothenic acid = 10 mg; Se = 0.1 mg; Vitamin A = 6000 IU; Vitamin B1 = 2 mg; Vitamin B12 = 0.01 mg; Vitamin B2 = 4 mg; Vitamin B6 = 2 mg; Vitamin D3 = 900 IU; Vitamin E = 40 mg; Vitamin K3 = 2 mg; Zn = 50 mg.

### Performance variables

The weight of the rabbits at the start and conclusion of the trial and the amount of feed consumed were recorded as follows:

$${\text{Weight gain}},{\text{ g}}/{\text{ rabbit }} = {\text{ W}}_{{\text{T}}} {-}{\text{ W}}_{0}$$

$${\text{Feed Conversion Ratio (FCR) = }}\frac{{\text{FI (g)}}}{{{\text{W }}_{{\text{T}}} {\text{ - W }}_{{0}} \, }}$$

where W_T_ = Final weight; W_0_ = Initial weight; FI = Feed intake.

### Sampling procedure

After 8 weeks of feeding, 5 rabbits/group were allocated for blood collection and slaughter. Blood samples were drawn without anesthesia from the lateral saphenous superficial vein of the back leg after wetting the fur with alcohol using a 1 ml syringe with heparin for hematological measurements or without anticoagulants to separate the serum. Heparin-treated blood was employed for hematocrit (Ht) quantification using microhematocrit tubes and rotary centrifugation (13,000 rpm for 5 min)^36^. Non-heparinized blood was centrifuged [3000 rpm undercooling (4 °C) for 10 min] to harvest serum. Hematological and biochemical blood indices were measured using CBC Micros ABX, France automatic analyzer with P500 kinetic & Quality control Diatron Q.C kits according to package guidelines. The liver and small intestine were separated on an ice layer, cleaned with regular saline solution (0.90%; pH 7.5), and subjected directly to the determination of hepatic antioxidants, intestinal structure, and digestive enzyme activities.

### Intestinal enzymes and histology assessment

Parts of the collected intestine (duodenum) were finely homogenized in freezing iced NaCl (0.86%) using VEVOR, FSH-2A device, and centrifuged at 8000 rpm for 5 min, 4 °C. The filtrate was employed for the colorimetric detection of amylase and lipase at A_714_ and A_540_^7^. Protease potency was measured using a non-specific protease vigor methodology utilizing casein^37^. For histological evaluation, samples (duodenum, jejunum, ileum) were fixed in a neutral buffered (10% formalin solution) for 72 h, dehydrated in rising grades of ethanol (60–100%), cleared in xylene, embedded in paraffin wax (24 h), and then sectioned with Rotary Microtome 2145, Leica Microsystems at a 3–5 μm in thickness.

### Hepatic antioxidants

Liver samples (5 rabbits/treatment) were finely homogenized in cold iced potassium phosphate buffer (pH 7.4, 10% w/v) using VEVOR, FSH-2A device, and centrifuged at 4 °C, 12,000 rpm for 10 min. The filtrate was employed for the colorimetric detection (Jenway UV–Vis spectrophotometer 7415, Staffordshire, UK) of superoxide dismutase (SOD), catalase (CAT), and glutathione peroxidase (GPx) at 550, 280, 412 nm using Biodiagnostic and research reagents, Dokki, Giza, Egypt.

### Statistical analysis

The study was designed in one-way ANOVA to compare the effects of the two algae species with two levels of supplementations in the feeds of New Zealand white rabbits. The rabbits were divided into five groups. The first group received a diet without either *A. platensis* or *C. vulgaris* (Control group, Ctrl). Conversely, the second group was given a diet with 300 mg/kg of *A. platensis* (Ap300), the third group a diet with 500 mg/kg of *A. platensis* (Ap500), the fourth group a diet with 300 mg/kg of *C. vulgaris* (Ch300), and the fifth group a diet with 500 mg/kg of *C. vulgaris* (Ch500). The data was examined using the IBM® SPSS® Inc., IL, USA program (IBM SPSS Statistics Ver. 26.0). The Shapiro–Wilk and Levene tests were employed to verify variance normality and homogeneity. The outcomes of the one-way ANOVA and Duncan's post hoc test were presented as a mean of three replicates with standard errors.

### Approval for animal experiments

The ethical committee of the Faculty of agriculture at Tanta University approved the experimental protocol and all methods in the present study for treating animals for scientific purposes (Approval No. AY_2019-2020_/Session 6/2020.01.13). All experiments were performed in accordance with relevant guidelines and regulations. Our reporting of research involving animals follows the recommendations of the ARRIVE guidelines.

## Results

### Chemical composition of the Algal supplements

**Figure 3:**
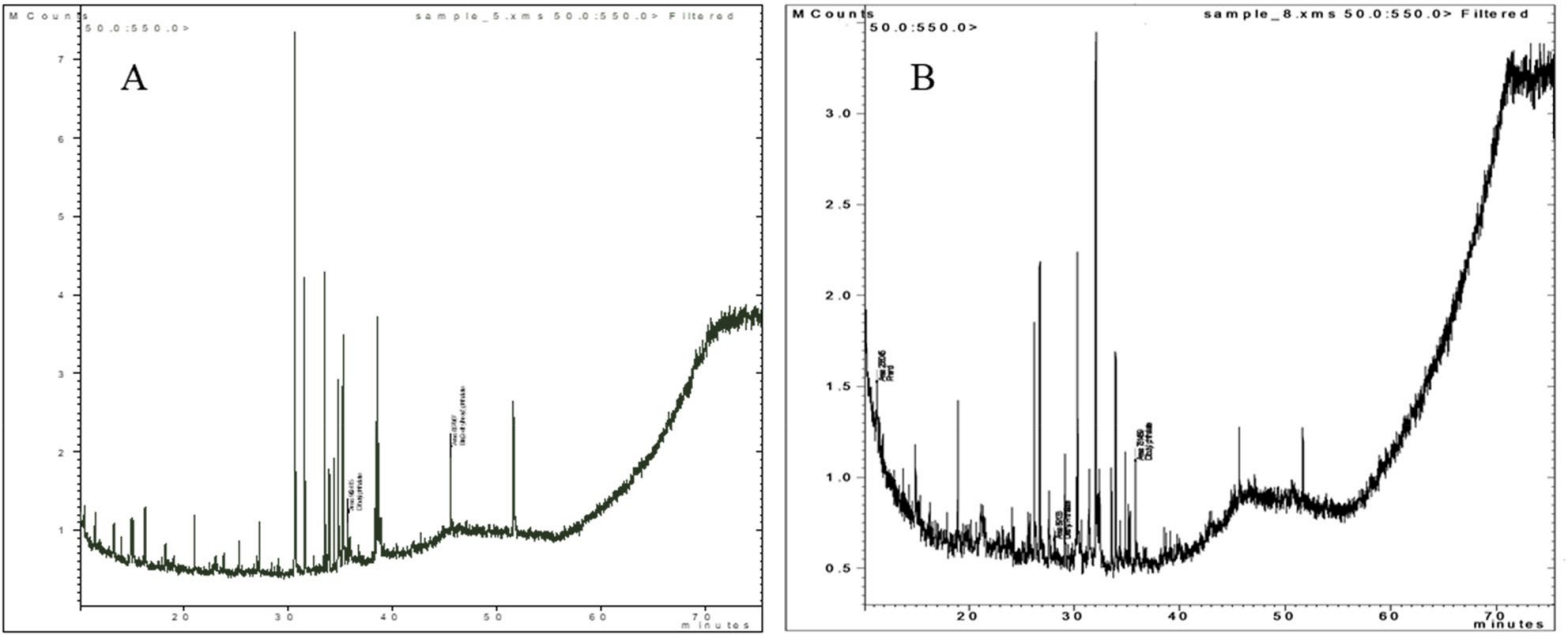
Chromatograms of Algal crude extracts. (**A**) *Arthrospira platensis* extract and (**B**) *Chlorella vulgaris* extract.

*Arthrospira platensis* dry biomass comprises 56.4 ± 3.3, 6.6 ± 0.6, and 26.2 ± 0.98% of protein, lipids, and carbohydrates, compared to *Chlorella vulgaris*'s proportions of 43.6 ± 2.4, 20.19 ± 1.2, and 23.8 ± 0.94%. A total of 25 active chemical compounds were characterized in the extracts of both algae. The identified chemical products with their retention time and % peak area of both extracts were shown in Table 3. The chromatograms of both extracts were shown in Fig. 3.

**Table 3:** Chemical constituents of the algal biomass and extracts.

Biomass proximate composition	*Arthrospira platensis*	*Chlorella vulgaris*		
Protein	56.4 ± 3.3%	43.6 ± 2.4%		
Lipids	6.6 ± 0.6%	20.19 ± 1.2%		
Carbohydrates	26.2 ± 0.98%	23.8 ± 0.94%		

GC–MS profiling of the algal extract	Molecular formula	Retention time, Min	Peak Area %	
			*Arthrospira platensis*	*Chlorella vulgaris*
Esters				
1,2-Benzenedicarboxylic acid, bis(2-methylpropyl) ester	C_16_H_22_O_4_	35.1	10.77	1.29
Pentadecanoic acid, 14-methyl-, methyl ester	C_17_H_34_O_2_	35.6	–	0.82
1,2-Benzenedicarboxylic acid, diisooctyl ester (Isooctyl phthalate)	C_24_H_38_O_4_	45.6	2.15	14.17
7-Hexadecenoic acid, methyl ester, (Z)-	C_17_H_32_O_2_	35.9	–	0.82
Hexadecanoic acid, methyl ester (Methyl palmitate)	C_17_H_34_O_2_	37	–	13.5
11-Octadecenoic acid, methyl ester	C_19_H_36_O_2_	38.8	–	0.45
Octadecanoic acid, 1-[(tetradecyloxy)carbonyl]pentadecyl ester	C_48_H_94_O_4_	25.1	2.15	22.3
Pentadecanoic acid, 14-methyl-, methyl ester	C_17_H_34_O_2_	35.3	6.46	–
Ethaneperoxoic acid, 1-cyano-1-[2-(2-phenyl-1,3-dioxolan-2-yl)ethyl]pentyl ester	C_19_H_25_NO_5_	35.7	7.32	–
1,2-Benzenedicarboxylic acid, diisooctyl ester	C_24_H_38_O_4_	39.1	2.15	–
Total			31.00	53.35
Fatty acid: Pentanoic acid, 4-methyl- (Isocaproic Acid)	C_6_H_12_O_2_	26	–	15.42
Fatty Alcohol: 3-Methyl-2-(3-methylpentyl)-3-buten-1-ol	C_5_H_10_O	27.5	1.72	6.4
Total			1.72	21.82
Hydrocarbon				
Eicosane	C_20_H_42_	32.5	1.29	–
4-Dodecene, (E)-	C_12_H_24_	26.1	–	17.32
Pentadecane	C_15_H_32_	33.8	–	6.29
Pentadecane, 7-methyl-	C_16_H_34_	30.8	18.96	5.1
Total			20.25	28.71
Ketone				
Propiophenone, 2'-(trimethylsiloxy)-	C_12_O_18_O_2_Si	21	1.72	–
1,4-Benzenediol, 2,6-bis(1,1-dimethylethyl)-	C_14_H_22_O_2_	18.2	13.94	–
Acetophenone, 2-chloro-	C_8_H_7_ClO	32	–	2.54
7,9-Di-tert-butyl-1-oxaspiro(4,5)deca-6,9-diene-2,8-dione	C_17_H_24_O_3_	33.4	4.3	3.4
Total			19.96	5.94
Steroids: Cholesterol	C_27_H_46_O	51.8	4.64	–
Terpenes				
3,7,11,15-Tetramethyl-2-hexadecen-1-ol	C_20_H_40_O	34.7	10.34	4.72
Phytol	C_20_H_40_O	38.6	3.44	1.2
3',8,8'-Trimethoxy-3-piperidyl-2,2'-binaphthalene-1,1',4,4'-tetrone	C_28_H_25_NO_7_	46.7	–	–
Squalene	C_30_H_50_	38.8	6.46	–
Total			20.24	5.92

In general, the identified chemical compounds belong to seven major chemical groups, including esters, fatty acids, fatty alcohol, hydrocarbons, ketone, steroids, and terpenes. *Chlorella vulgaris* extract contains more esters (53.35%), fatty compounds (21.82), and hydrocarbons than *Arthrospira platensis* extract (31.00, 1.72, and 20.25, respectively). Meanwhile, *Arthrospira platensis* extract contains more ketone (19.96%), cholesterol (4.64%), and terpenes (20.24%) than* Chlorella vulgaris* extract (5.94, 0.00, and 5.92%, respectively).

### Performance variables

Table 4 shows the growth and nutrient efficiency of New Zealand white rabbits fed experimental diets for 8 weeks. Rabbits fed the basal diet exhibited the lowest final weights and weight gains and the highest feed conversion ratio, which improved noticeably with algae addition, particularly with Ap500, Ch300, and Ch500. Feed intake did not change with treatments except for the high level of *Chlorella vulgaris* (Ch500), which showed the lowest FI value.

**Table 4:** Growth, nutrient efficacy variables, and efficiency of intestinal enzymes of New Zealand white rabbits (initial weight = 665.93 ± 15.18 g) fed experimental diets for 8 weeks.

Parameters	Control	*Arthrospira platensis*		*Chlorella vulgaris*	
		Ap300	Ap500	Ch300	Ch500
Initial body weight, g/rabbit	662.67 ± 50.40	671.00 ± 32.45	649.33 ± 35.62	679.67 ± 16.80	667.00 ± 51.63
Final body weight, g/rabbit	1906.00 ± 77.66^b^	2092.00 ± 57.36^ab^	2236.33 ± 64.92^a^	2148.67 ± 25.39^a^	2205.00 ± 62.96^a^
Body weight gain, g/rabbit	1243.33 ± 27.76^b^	1421.00 ± 81.64^ab^	1587.00 ± 49.56^a^	1469.00 ± 41.47^ab^	1538.00 ± 114.53^a^
Feed intake, g/rabbit/56 day	6114.00 ± 66.11^a^	6107.00 ± 55.42^a^	5726.00 ± 109.02^ab^	5891.67 ± 187.72^a^	5385.33 ± 144.46^b^
Feed conversion ratio	4.92 ± 0.7^a^	4.32 ± 0.22^b^	3.62 ± 0.17^c^	4.01 ± 0.06^bc^	3.55 ± 0.34^c^
Amylase	19.44 ± 1.83	23.10 ± 1.49	22.71 ± 2.93	25.34 ± 3.06	21.56 ± 2.49
Lipase	17.71 ± 1.47^b^	26.95 ± 1.57^a^	26.33 ± 0.97^a^	25.17 ± 2.11^a^	25.90 ± 1.66^a^
Protease	15.02 ± 2.07^b^	28.43 ± 2.48^a^	26.96 ± 2.58^a^	26.88 ± 2.42^a^	25.50 ± 2.15^a^

Values are means ± standard errors. Numbers with unique letters vary statistically (*P* < 0.05).

### Intestinal efficiency

**Figure 4:**
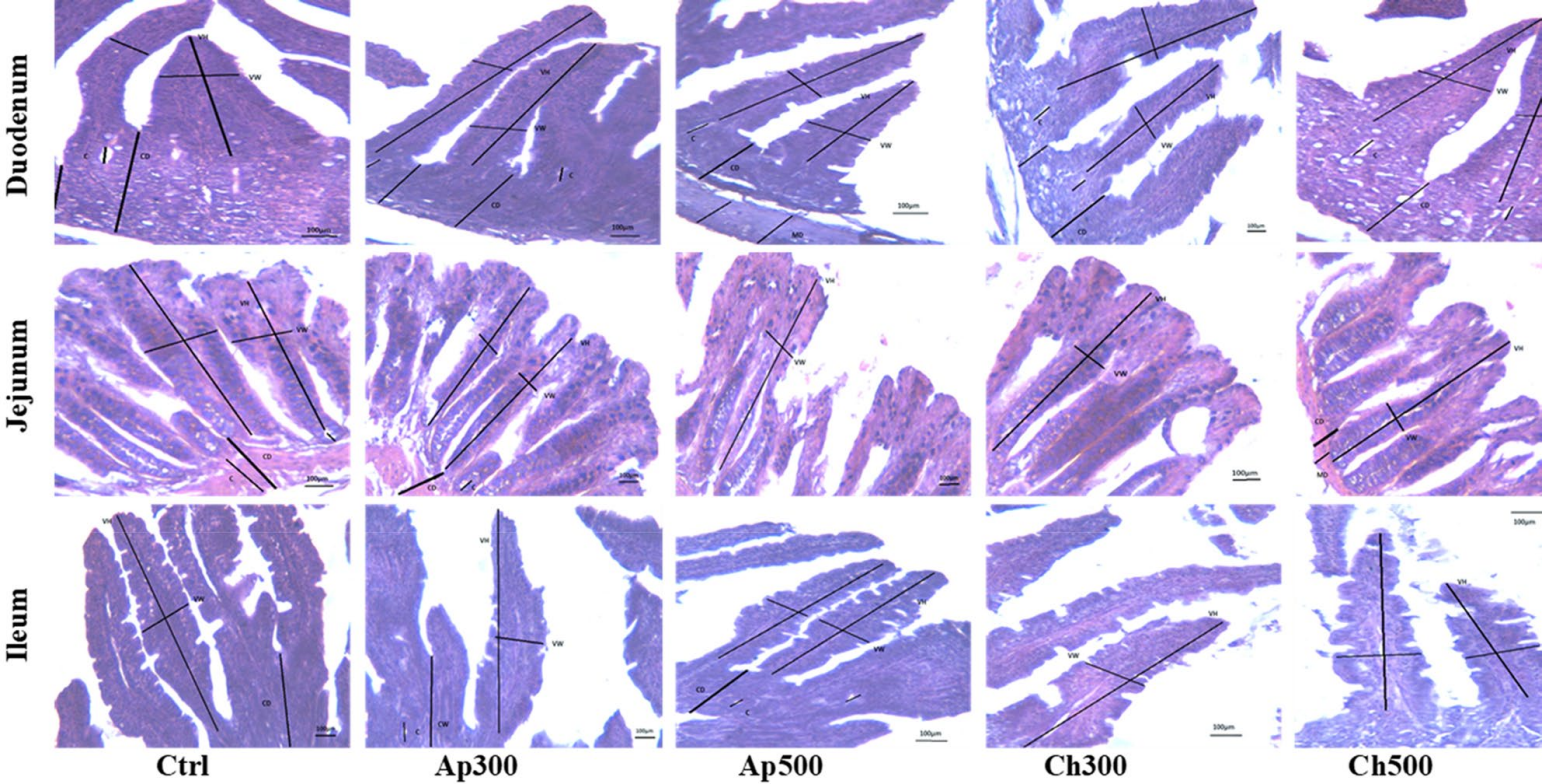
Intestinal structure (duodenum, jejunum, ileum, H&E = 40 X) of New Zealand white rabbits fed experimental diets for 8 weeks. Ctrl = the control group; Ap300 and Ap500 = *Arthrospira platensis* inclusion levels at 300 and 500 mg/kg; Ch300 and Ch500 = *Chlorella vulgaris* inclusion levels at 300 and 500 mg/kg.

Figure 4 shows the intestinal structure of New Zealand white rabbits fed experimental diets for 8 weeks. All rabbit groups showed intact and normal intestinal structures with no pathological alterations such as degeneration, necrosis, hemolysis, edema, congestion, hemorrhages, and hypertrophy. The efficiency of intestinal enzymes is shown in Table 4. A remarkable enhancement in the efficiency of lipase and protease occurred in algal groups compared to the control, while the efficiency of amylase did not change between the experimental groups.

### Blood health

Table 5 exhibits the blood profile of New Zealand white rabbits after 8 weeks of feeding trial. Hematological indicators comprising hematocrit (Ht), hemoglobin (Hb), red blood cells (RBCs), and white blood cells (WBCs) showed non-significant variation with dietary treatments. Similarly, serum biochemistry displayed no alteration in glucose, triglyceride, alanine transaminase (ALT), and aspartate transaminase, while a significant alteration occurred in total protein and total cholesterol. Rabbits treated with *Arthrospira* (Ap300 and Ap500) and *Chlorella* (Ch300 and Ch500) exhibited higher total protein and lower total cholesterol compared with the reference group. The lowest level (*P* < 0.05) of cholesterol was found in the blood of rabbits given a high level of *Arthrospira* (Ap500) and both levels of *Chlorella* (Ch300 and Ch500).

**Table 5:** Blood profile of New Zealand white rabbits fed experimental diets for 8 weeks.

Parameter	Control	*Arthrospira platensis*		*Chlorella sp.*	
		Ap300	Ap500	Ch300	Ch500
Hematocrit (Ht, %)	28.14 ± 3.76	28.75 ± 2.32	30.80 ± 3.00	30.70 ± 3.86	30.81 ± 2.09
Hemoglobin (Hb, g/dl)	6.78 ± 0.30	6.70 ± 0.56	6.67 ± 0.61	6.80 ± 0.42	6.75 ± 0.33
Red blood cells (RBCs, 10^6/^µl)	1.83 ± 0.14	2.26 ± 0.13	2.33 ± 0.34	2.52 ± 0.32	1.95 ± 0.15
White blood cells (WBCs, 10^3/^µl)	61.96 ± 5.09	81.89 ± 7.34	77.04 ± 6.70	75.03 ± 5.02	73.20 ± 5.05
Glucose (mg/dl)	95.10 ± 7.89	96.44 ± 9.19	74.98 ± 3.18	88.03 ± 16.58	91.30 ± 11.08
Total protein (TP, g/dl)	5.04 ± 0.23^b^	6.65 ± 0.13^a^	6.68 ± 0.26^a^	6.58 ± 0.08^a^	6.43 ± 0.14^a^
Total cholesterol (T-Chol, mg/dl)	137.01 ± 3.66^a^	119.86 ± 2.58^b^	114.69 ± 4.29^bc^	107.96 ± 2.05^c^	105.85 ± 0.80^c^
Triglyceride (mg/dl)	100.18 ± 3.21	102.73 ± 1.75	95.79 ± 6.47	97.64 ± 5.99	98.53 ± 8.30
Alanine transaminase (ALT, IU/l)	29.43 ± 2.70	32.91 ± 3.44	31.42 ± 2.81	33.29 ± 3.38	28.12 ± 3.18
Aspartate transaminase (AST, IU/l)	57.97 ± 4.92	61.92 ± 3.35	54.54 ± 7.60	59.80 ± 3.52	55.64 ± 4.46

Values are means ± standard errors (n = 3 /replicate). Numbers with various letters differ statistically (*P* < 0.05).

### Hepatic antioxidants

**Figure 5:**
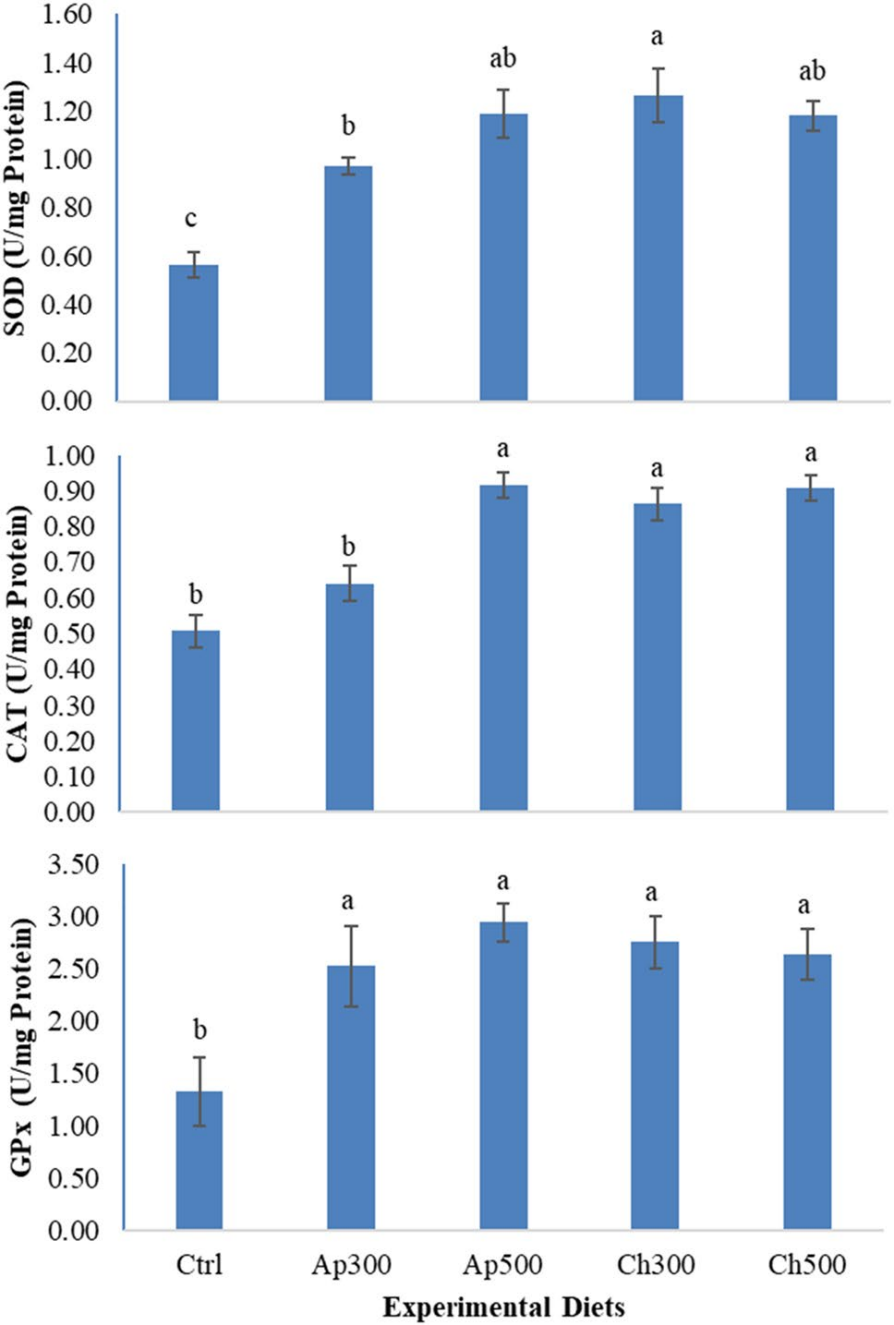
Hepatic superoxide dismutase (SOD), catalase (CAT), and glutathione peroxidase (GPx) activities in New Zealand white rabbits fed experimental diets for 8 weeks. Ctrl = the control group; Ap300 and Ap500 = *Arthrospira platensis* inclusion levels at 300 and 500 mg/kg; Ch300 and Ch500 = *Chlorella vulgaris* inclusion levels at 300 and 500 mg/kg.

Figure 5 displays the hepatic superoxide dismutase (SOD), catalase (CAT), and glutathione peroxidase (GPx) activities in New Zealand white rabbits after 8 weeks of feeding trial. Rabbits fed the basal diet exhibited the poorest antioxidant potency (SOD, CAT, and GPx). The best GPx existed in all groups fed algal diets, while the favored SOD and CAT efficacy appeared at higher *Arthrospira* (Ap500) and both levels of *Chlorella* (Ch300 and Ch500).

## Discussion

Stimulating the maximum production of animals necessitates specific procedures to ensure quantity, quality, and animal health^7^. Nutraceuticals feed additives in the animal production business as natural substitutes for antibiotics have risen in importance^6^. Rabbit production is an appropriate agricultural investment because of its low production costs, superior fertility, short generation intervals, and ability to utilize a range of forages^1^.

Results of growth variables showed that rabbits fed the basal diet exhibited the poorest performance, which improved noticeably with algae addition, particularly with Ap500, Ch300, and Ch500 (Table 4). The high nutritional value of algae may be one of the reasons for the enhanced performance in animals fed with algae supplements. In this sense, Mahmoud et al.^18^ found that soybean substitution by *A. platensis* at levels of 20, 40, and 60% in rabbit feed did not show any negative outcomes and maintained indicators of growth, health, and meat quality. Furthermore, Seyidoglu et al.^26^ found an enhancement in the immune system of growing rabbits with *A. platensis*-diets. In contrast, Gerencsér et al.^38^ assumed that *Arthrospira* (5%) and thyme (3%), either alone or in combination, did not substantially alter the growth or health of growing rabbits. *Chlorella* has been suggested to boost the performance and health of animals^17,30,39^. In a previous study, Hassanein et al.^40^ compared the influence of *Arthrospira* (*Spirulina*)* platensis* and *Chlorella vulgaris* at levels of 0.75 and 1.5 g/kg diet on growing New Zealand white rabbits and concluded that both levels of *A. platensis* improved the growth and reduced liver enzyme, cholesterol and total lipids contents in serum in comparison with *Chlorella vulgaris* supplements. Moreover, An et al.^39^ demonstrated that adding 0.15% dried *Chlorella vulgaris* powder to Ross broiler chicks' feed considerably improved growth, blood cell counts, and declined total lipids in serum. According to Abdelnour et al.^30^, adding 1.0 g of *Chlorella vulgaris* to the diet of growing New Zealand white rabbits could boost their immunological and antioxidant health, as well as reduce blood lipid accumulation. Despite these valuable results and to the authors' best knowledge, so far there are no planned studies that have compared the potentials of Arthrospira and Chlorella on intestinal histology, digestive enzyme potency, and hepatic antioxidants of New Zealand white rabbits. Consequently, the current trial was designed to cover these parameters.

Likewise, the improvement in weight with algae supplements may be linked to a change in the feed conversion ratio (**↓** FCR). The detected reduction in FCR may be linked with the amended intestinal efficiency (Table 4), particularly digestive enzymes (lipase and protease). Several studies have demonstrated that adding algal biomass or extracts improves growth and nutrient use. In *Arthrospira* impacts regard, Alazab et al.^20^ found that adding *Spirulina platensis* (SP) to the diet of growing rabbits at a level of 0.6 g/kg diet resulted in considerably better growth performance parameters and enhanced feed conversion ratio in comparison to those provided the low level (0.3 g/kg diet) or those fed a basal diet. Moreover, Aladaileh et al.^21^ highlighted that exogenous supplementation of SP enhanced the growth traits of rabbits subjected to Pb. In addition, Peiretti and Meineri^22,23^ demonstrated that rabbits receiving *Arthrospira* at a level of 10% exhibited higher feed consumption. Regarding the effect of *Chlorella*, Sikiru et al.^31^ noted that dietary implementation of the *Chlorella vulgaris* amidst 200 and 500 mg/kg diet considerably raised the rabbits’ weights without substantial alteration in feed intakes, but substantially enhanced feed to gain ratio. In another study by Sikiru et al.^32^ on New Zealand white rabbits, a significant positive boost in the final body weight and feed intake with the addition of *Chlorella vulgaris.* In contrast to the findings of the current study, no alteration was observed in the growth aspects with the dietary incorporation of *Arthrospira* (*Spirulina*)^22–24,38^ or *Chlorella*^30^ and this may be due to the different conditions of the experiment.

Blood status is a precise sign of the welfare and health status of animals, hence are direct reflectors of stressors and external stimuli^41^. Hematological indicators and serum biochemistry showed non-significant variation except for serum total protein and total cholesterol (Table 5). Rabbits treated with *Arthrospira* and *Chlorella* had higher total protein (TP) and lower total cholesterol than the reference group. The higher levels of TP in rabbits fed algae may suggest an improvement in rabbit health. In this context, Hassan et al.^19^ reported an enrichment in plasma total protein in rabbits provided a diet enhanced with Zn-Se- rich *Spirulina* compared to the reference group. A similar improvement in glycoprotein appeared with *Chlorella* treatment^33^. The hypocholesterolemic effect of algae could explain the lower cholesterol levels associated with supplementation. In line with the present results, Cheong et al.^27^ suggested that spirulina consumption can reduce hypercholesterolemic atherosclerosis by lowering total serum cholesterol in New Zealand White rabbits. Also, Hassan et al.^19^ found low levels of total cholesterol, LDL- and VLDL-cholesterol in Se-rich *Spirulina* and Zn-Se- rich *Spirulina* groups of New Zealand White male rabbits. Similar impacts on cholesterol were reported with *Chlorella* incorporation. In this regard, Abdelnour et al.^30^ found a reduction in serum VLDL in the *Chlorella*-treated groups relative to those in the control group.

The oxidative state of the animal is positively related to its immunity and wellbeing^42^. Oxidative stress is caused by an imbalance in the generation and clearance of reactive oxygen species (ROS)^43^. Several enzymes in the oxidative system, such as SOD, CAT, and GPx aid in the elimination of ROS and the maintenance of cell homeostasis^44^. In the present trial, algae dietary application mediates a substantial rise in SOD, CAT, and GPx activities. This may be due to the unique compositions of *Arthrospira* and *Chlorella* that are rich in effective compounds with an antioxidant impact, e.g., minerals, vitamins, β carotene, β-glucan, linolenic acid, tocopherols, phycocyanin, flavonoids, and phenols. Similar interpretations were reported for New Zealand White rabbits fed *Arthrospira* by Hassan et al.^19^ or* Chlorella* by Abdelnour et al.^30^. Several studies have found enhanced antioxidant enzymes in rabbits fed *Arthrospira*^21,28,29^ and *Chlorella*^30,32^.

The content of active chemicals in feed additives is mostly responsible for their beneficial effects. The overall results indicated that including *Chlorella* surpassed *Arthrospira* additives in New Zealand white rabbit feeds. These results indicated superior improvements in the growth performance, feed efficiency, and intestinal and blood health of New Zealand white rabbits fed *Chlorella*. These observations could be associated with the content of *Chlorella* and its effect on intestinal health and body immunity in rabbits. The GC–MS analysis of the crude extracts of the two algae showed the presence of 25 chemical substances with known favorable bioactivity on rabbits^19–21,31^ and humans^45^. It is challenging to explain the effects of algae dietary supplements at the level of a single ingredient because algal extracts contain a significant number of active compounds and the best strategy is to classify them into major categories. *Arthrospira* exceeded *Chlorella* in its content of ketone, cholesterol, and terpenes, but *Chlorella* surpassed *Arthrospira* in its content of esters, fatty compounds, and hydrocarbon. Both extracts contain the majority of the active chemicals, albeit in different amounts which amply explain the convergence of the impacts supporting the performance and wellbeing of New Zealand white rabbits. In this context, phytol is a diterpene compound found in almost all crude extracts of the used algae and known for its anticancer and antioxidative properties^46^. The hydrocarbon pentadecane and the fatty acid Pentanoic acid, 4- methyl- are known for their antimicrobial activity^47^ and antitumor activity^48^, as well as a growth promoter^49^.

## Conclusions

The present study sheds light on the potential of algae feed additives (*Arthrospira platensis* VS* Chlorella vulgaris*) on the performance and wellbeing of New Zealand white rabbits. Incorporating *Arthrospira platensis* at 500 mg/kg diet or *Chlorella vulgaris* at levels of 300 and 500 mg/kg diet improved growth, nutrient aspects, intestinal enzyme efficiency, blood health, and antioxidants of New Zealand white rabbits. It will be vital for rabbit production in the future to monitor molecular responses to external feeds and/or supplements and to concentrate on obtaining a precise nutritional formula for algal biomass in rabbit feeding without compromising performance or health.

## Data Availability

The datasets used and/or analysed during the current study available from the corresponding author on reasonable request.
